# Vitiligo in a Patient Treated with Interferon Alpha-2a for Behçet's Disease

**DOI:** 10.1155/2012/387140

**Published:** 2012-08-16

**Authors:** Esra Guney, Gulunay Akcali, Betul Ilkay Akcay, Cihan Unlu, Gurkan Erdogan, Tahir Kansu Bozkurt, Huseyin Bayramlar

**Affiliations:** Ophthalmology Clinic, Umraniye Research and Education Hospital, Adem Yavuz Caddesi, Ümraniye, İstanbul, Turkey

## Abstract

Behçet's disease (BD) and vitiligo are diseases of unknown etiology. Interferon (IFN) alpha therapy is commonly used in Behçet uveitis. Interferon treatment in various diseases have also been observed causing certain autoimmune diseases such as vitiligo because of its immunomodulatory activity. The association between IFN therapy and vitiligo has been reported in the literature. We report a 21-year-old man with BD in whom vitiligo occurred during IFN treatment. To the best of our knowledge, this is the first reported case of such an association.

## 1. Introduction

Behçet's disease (BD) is a chronic, relapsing, vascular inflammatory disease of unknown etiology affecting all sizes of arteries and veins. Ocular involvement in these patients are common and the typical form of involvement is a bilateral nongranulomatous panuveitis and retinal vasculitis. The exact cause of BD still remains unknown, however, current evidence suggests that a complex interplay of genetic and environmental factors triggers an autoimmune process in genetically predisposed individuals [1].

Interferons (IFN) are part of the nonspecific immune system and are induced at an early stage by pathogens such as viruses, bacteria, parasites, or tumor cells. They have antiviral, antimicrobial, antitumor, and immunomodulatory actions [2]. Due to its effects, IFN therapy has gained popularity in the treatment of Behçet's disease in the last decade. Several studies have shown consistent benefit with the use of IFN in the treatment of Behçet's uveitis [3–5]. A wide array of adverse effects of IFN alpha therapy has been described. The major side effects of IFN alpha therapy in Behçet' disease are flu-like symptoms skin lesions such as psoriasis development of autoantibodies, thyroid hormone imbalance, severe depression, and leukopenia [3–6]. Furthermore necrosis and vasculitis at the injection site, xerosis, pruritus, urticaria, alopecia, psoriasis, ptyriasis rosea, lichen planus, eosinophilic fasciitis, and vitiligo are the most common adverse skin reactions due to IFN treatment [2, 7, 8].

Vitiligo is a common, idiopathic, progressive, acquired depigmenting skin disorder in which some or all the melanocytes are selectively destroyed in the hypomelanotic areas [9, 10]. The destruction is thought to be due to an autoimmune problem, however, multiple immunological, neurological, and genetic components have been considered in the pathogenesis of the disease [11].

We report a patient in whom vitiligo occurred during IFN alpha-2a therapy for Behçet panuveitis. To the best of our knowledge, this is the first report of such an association.

## 2. Case Report

We administered IFN alpha-2a to a 21-year-old man with bilateral Behçet panuveitis. He fulfilled the classification criteria of the International Study Group for Behçet's disease [12]. Except for his eye lesions, he had recurrent oral ulcerations and, recurrent genital ulcerations. He had previously received high-dose corticosteroids and conventional immunosuppressive treatment with azathioprine and cyclosporine. IFN alpha-2a therapy was initiated during his remission period to prevent the side effects of high-dose corticosteroids.

**Figure 1:**
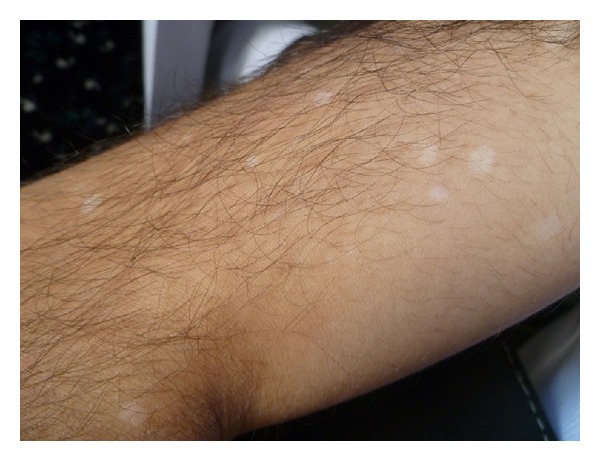
Vitiligo lesions on the upper arm of patient.

Immunosuppressive agents and oral corticosteroids were discontinued before initiation of IFN alpha-2a therapy. The initial dose of IFN alpha-2a was 6 million units per day (MU/day), subcutaneously, which was tapered to 3 MU/day after 15 days. The patient tolerated the therapy well and no further uveitis attacks were observed during the treatment. However, after 2 months of therapy, he developed small, round, depigmented areas bilaterally on the upper arms, around the IFN alpha-2a injection sites (Figure 1). Diagnosis of vitiligo has been confirmed for dermatologist consultation. The patient had no family history of vitiligo. Topical corticosteroid treatment was initiated for his vitiligo and the maintenance dose of IFN alpha-2a was adjusted to 3 MU three times weekly. His condition did not change in the following 2 months.

## 3. Discussion

Many cases of vitiligo associated with IFN treatment have been reported [13–15]. The exact relationship between the treatment and this autoimmune phenomenon is still obscure. It can be hypothesized that stimulation of autoantibodies against melanocytes and/or cytotoxic T-cell activation by IFN therapy can lead to vitiligo development [16]. It has also been shown that the presence of autoantibodies prior to IFN therapy increases the risk of developing autoimmune disorders once IFN is initiated [17]. Interferon has many functions on the immune system, including modulation of immunoglobulin production; inhibition of T suppressor cell function and stimulation of T-cell cytotoxicity, monocyte, and macrophage and natural killer cell activity [13, 18].

It is well known that vitiligo is associated with various autoimmune disorders and autoimmunity is supposed to play a role in its pathogenesis [19]. The frequency of autoimmune thyroid disease is increased among vitiligo patients and their first-degree relatives [20] and the frequency of vitiligo is higher among patients with Hashimoto's thyroiditis and Graves' disease [21]. Oran et al. reported that the frequency of vitiligo was not increased among patients with BD [22]. They claimed that traditional autoimmune mechanisms might not be operative in BD. However, Borlu et al. reported two brothers who exhibited coexisting BD and vitiligo. They attributed this association to some common features of both diseases, including T-cell activation and higher levels of autoantibodies [23]. Nevertheless, they did not deny the possibility of a coincidence in their case. There have not been many reports about an association between BD and vitiligo. However, there are several case studies published in the literature about vitiligo occurring during IFN treatment [13–15]. Taken together, the occurrence of vitiligo during the course of BD in our case suggests that IFN therapy, rather than BD, may play a role in the development of vitiligo. Anbar et al. reported a case with vitiligo occurring at the site of IFN injection. They claimed that vitiligo occurred as a result of local immune response against melanocytes at the sites of IFN injection [24]. In our case, that kind of local immune reaction may be responsible for vitiligo formation at IFN injection sites. Many dermatological side effects have been reported with IFN. Some, but not all, of these side effects were immune-mediated. Psoriasis, pemphigus, vitiligo, and alopecia were immune-mediated side effects [17]. Most of the side effects associated with IFN are considered to be dose-dependent [2, 7]. In a previous study, the occurrence of vitiligo with IFN therapy for viral hepatitis was reported in a series of eight cases [14]. However, it is not clear whether the IFN dose is correlated with the development or exacerbation of vitiligo. In our case, the skin lesions of the patient did not improve after tapering the dose of IFN alpha-2a. Occurrence of vitiligo has previously been reported in patients treated with IFN for hepatitis C, hepatitis B, and melanoma [13, 14, 24]. To the best of our knowledge, this is the first reported case of vitiligo in a BD patient treated with IFN. This case is an important reminder of the potential side effects of IFN and the need to warn patients about them before initiating IFN therapy.
